# Prevalence of children living with non-biological parents and its determinants among children under 18 in Ethiopia: a multi-level mixed effect analysis

**DOI:** 10.3389/fpubh.2024.1420002

**Published:** 2024-12-19

**Authors:** Berhan Tekeba, Alebachew Ferede Zegeye, Tadesse Tarik Tamir

**Affiliations:** ^1^Department of Pediatrics and Child Health Nursing, College of Medicine and Health Sciences, School of Nursing, University of Gondar, Gondar, Ethiopia; ^2^Department of Medical Nursing, College of Medicine and Health Sciences, School of Nursing, University of Gondar, Gondar, Ethiopia

**Keywords:** children under 18, non-biologic parent, orphan, living arrangement, Ethiopia

## Abstract

**Introduction:**

The absence of a biological parent from a child's existence had a negative impact on the child's growth, socialization, psychological wellbeing, and economic productivity. Developing nations like Ethiopia experience a huge number of orphans and family-unbounded children. But the exact figure has not been reported yet at the national level recently. Thus, this study aimed to assess the magnitudes and determinants of children living with non-biologic parents in Ethiopia.

**Methods:**

Secondary data analysis was conducted based on the demographic and health survey data conducted in Ethiopia in 2016. A total weighted sample of 41,884 children under the age of 18 from 5 years preceding the survey was included in this study. A multi-level logistic regression model was used to identify the determinants of children living with non-biologic parents. The adjusted odds ratio at 95% Cl was computed to assess the strength and significance of the association between explanatory and outcome variables. Factors with a *p* < 0.05 are declared statistically significant.

**Results:**

The prevalence of children living with non-biologic parents in Ethiopia was 17.58% (95% CI, 17.22–17.95). Being an orphan (AOR = 4.57, 95% CI: 2.40–7.25), being in first birth order (AOR = 8.22, 95% CI: 6.31–9.17), being from a household lacking formal family structure (AOR = 8.60, 95% CI: 6.20–12.30), and being from a female-headed household (AOR = 3.43, 95% CI: 2.65–4.43) were individual-level factors that were significantly associated with children living with non-biologic parents. Being a rural resident (AOR = 1.94, 95% CI: 1.23–3.08) and having a high community poverty level (AOR = 1.25, 95% CI: 1.01–1.75) were community-level determinants of children living with non-biologic parents.

**Conclusion:**

According to this study, a significant proportion of children live with non-biological parents in Ethiopia. Thus, policymakers, health planners, and implementers need to give special attention to children from rural communities, orphans, firstborn children, and broken families. In addition, efforts shall be made to empower women and, in the long run, improve the economy of the community.

## Introduction

Children living with a non-biologic partner are children whose parents have died or are not living with them for any reason. Globally, while it is difficult to pinpoint the exact number of children who have lost one or both parents for a variety of causes, the figure is thought to be in the millions.^1^ Globally, approximately one in ten (10%) children lives without biological parents. However, it varies across countries; for instance, in high-income countries like the USA, about 2.5% of children live in adopted households, whereas in Sub-Saharan Africa, the prevalence reaches as high as 30% due to factors related to HIV/AIDS, poverty, and conflict.^2^ In Ethiopia, according to a United Nations International Children Emergency Fund (UNICEF) report, nearly half (46.05%) of the population were children under the age of 18 (1). The country is characterized by a high fertility rate (4.0%) and rapid population growth (2). Many children live without their biological parents due to several socio-economic environmental challenges, including poverty, displacement, disease, and child marriage in Ethiopia.^3^

There are numerous, frequently mentioned reasons why children split up from their parents. Children not living with their biological parents were resulted from high population growth, poverty, parental death, and illegal adoption in Ethiopia (see text footnote 3). Poverty is arguably the most frequent cause of parental separation worldwide. Aside from its negative impacts, poverty damages families and communities as a whole by leaving children behind. Additional factors that can lead to orphanhood and separation from biologic parents include drug misuse by parents, natural catastrophes, poverty, HIV/AIDS, drought, war, hunger, family breakdown, parent death, military deployment, mental illness of the parent, cognitive impairment of parents, conflict, violence, and parental relocation (3–5).

Several studies have indicated that parents are crucial to their children's healthy development and growth. Compared to children who live with a biological parent, those who grow up and live with their non-biological parent's exhibit less self-control behaviors, such as aggressiveness, being less confident, and having a poor attitude (7). In addition, these children are prone to missed education, food insecurity, poor health, teenage pregnancy, socio-economic disadvantage, disruption, instability, multiple sexual partners, attention deficit hyperactive disorder (ADHD), exploitation, violence, discrimination, and low academic achievement in school (7–10).^4^,^5^,^6^ The government of Ethiopia made efforts to protect these children by adopting constitutions and laws to protect harm against these vulnerable children (6); however, many of the relevant child rights rules and regulations have not yet been implemented to a satisfying degree in the country (7). Furthermore, the problem persists in the country secondary to drought, ongoing civil war, natural disasters, and inflation. Thus, examining the social, economic, and health burden on these children and identifying determinants helps responsible bodies develop policies and interventions to support these children so that they can access resources and appropriate care.

Ethiopia, as a nation, had a higher proportion of children under the age of eighteen. As previously reported, approximately 11% of children did not live with their biological parents. Recent estimates suggest a higher number of parent-unbounded children in the country, but the exact figure remains unknown. In addition, the factors responsible for the unattachment of children to their biological parents are scarcely studied in the country. Therefore, this study aimed to update the prevalence of children living with non-biologic parents and its predictors in Ethiopia using 2016 EDHS as a data source.

## Objectives

### General objective

To determine the prevalence of under-eighteen children living with non-biologic parent in Ethiopia.

### Specific objective

To identify factors associated with under-eighteen children living with non-biologic parent in Ethiopia.

## Methods

### Study area

Ethiopia is a country located in the Horn of Africa. The country located at geographical coordinates of 9.145° N latitude and 40.48° 97° East longitude (see text footnote 4). The nation's overall surface area is thought to be 1,126,829 km^2^. Its neighbors are Djibouti, Eritrea, Kenya, Somalia, South Sudan, Sudan, and Somaliland. There are 13 administrative regions in Ethiopia, namely Tigray, Afar, Amhara, Gambela, Benishangul Gumuz, Harari, Oromia, Somalia, Southern Nation Nationalities and Peoples Region (SNNP), Sidama, South West Peoples (a recently added), and two city administrations (Addis Ababa and Diredawa). Each of the country's 11 administrative regions and two administrative cities are organized into zones, districts, towns, and kebeles (the smallest administrative units). However, the Ethiopian demographic and health survey data set used in this study does not provide data for the Sidama region and South West Peoples region. More than 84% of the population of Ethiopia resides in rural areas (8). Ethiopia had a deeply rooted kinship arrangement of cultural norms that emphasize collective child rearing and a community-based support system, particularly during periods of crises such as drought, parent loss, conflict, and economic hardships.

### Data source

We used data from the Ethiopian Demographic and Health Survey 2016, conducted from January 18, 2016 to June 27, 2016. It is conducted every five intervals, which was the fourth survey conducted nationally. The data were collected from nine regional states and two city administrations. The Ministry of Health and Ethiopia's Central Statistical Agency (CSA) worked together to collect the data.

### Sampling procedure

A two-stage stratified cluster sampling technique was applied. In the first stage, 645 enumeration areas (202 urban and 443 rural EAs) were selected, with probability sampling proportional to the size of the EAs. Secondly, households where data is collected are selected. A total of 41,884 weighted children were extracted from EDHS in 2016. The full EDHS 2016 report, which included information on the sampling process as well as general data collection, is found elsewhere (see text footnote 5). Children living arrangement response which had a missing value for the outcome were excluded from the study.

### Study setting

#### Sample and populations

The source population was all children living with their non-biologic parent in Ethiopia 5 years preceding the survey in the enumeration area of sampling clusters, whereas all selected children in the household lived with their non-biologic parent in Ethiopia 5 years preceding the survey in the enumeration area of sampling clusters. A two-stage stratified cluster sampling technique was employed. Stratification was done by separating each region into urban and rural areas. In stage one, 645 EAs were selected using probability sampling. In the second stage, households were selected systematically in each enumeration area. Then, children were selected using a PR file, and important variables were selected from the data set.

#### Study outcome variables

The outcome variable of this percentage of De Jure children under the age of 18 living with a non-biologic parent was dichotomized as “Yes = 1” for children who live with a non-biologic parent and “No = 0” for children who live with their biologic parent. The outcome was determined by using Demographic and Health Survey Data, under children living arrangements and orphaned sub-section, the number of children under eighteen, i.e., not living with biologic parents, including (i) both parents alive but not living with them. (ii) Only one parent is alive but does not live with either; (iii) both parents are dead and live with other families (see text footnote 6). By summing up the above categories, the proportion of children living with non-biologic parents was determined.

#### Independent variables

Both individual and community-level factors were reviewed from different literatures, and these include educational level, child sex, orphanage, history of family death, non-normal family structure, sex of the household, birth order, the number of under-five in the household, and the household wealth quintile, which belong to individual-level factors. Region, place of residence, community education, and community wealth status were community-level factors aggregated from individual-level factors.

Wealth indexes were categorized as poor, middle, and rich. Education status was categorized as having no formal education and having formal education by combining paternal and maternal education levels.

#### Community level variables

Community-level women's illiteracy and community-level poverty were aggregated from individual women's education, and wealth index, respectively. Since the aggregate values for all generated variables have no meaning at the individual level, they were categorized into groups based on median values. Median values were used to categorize as high and low because all aggregated variables were not normally distributed. Similar procedures were applied to all aggregate variables.

Regarding the analysis of aggregation, first the individual-level variables were re-categorized and cross-tabulated with the cluster variables using Stata version 17. Then, the proportions of ANC use, no education, and poor wealth index categories for each variable proportion were computed using Microsoft Excel 2010. Then, the proportion of each variable result was imported into Stata and combined with the original Stata using a one-to-many variable combination. Finally, we categorized the proportion of each variable as high or low based on the mean proportion.

### Data collection procedure

The DHS Program granted us permission to collect and use the data from http://www.dhsprogram.com for this study after we asked permission. Before releasing the data collected from the DHS to the public, participant identification was erased, and institutional ethical approval was waived to ensure compliance with the rules governing the protection of human beings.

### Data management and model selection

#### Model building for multi-level analysis

In DHS, data variables are nested by clusters, and those within the same cluster show more similarities than those with separate clusters. Thus, using the traditional logistic regression model violates the assumptions of independent observation and equal variance across clusters. Therefore, a multi-level logistic regression analysis was employed in this study in order to account for the hierarchical nature of DHS data. A bivariate multi-level logistic regression model was employed in the study to identify factors associated with children living with non-biologic parents. In the analysis, four models were fitted. The first (null) model contains only the outcome variables to test random variability and estimate the intra-cluster correlation coefficient (ICC). The second model contains individual-level variables; the third model contains only community-level variables; and the fourth model contains both individual-level and community-level variables (9). Due to the hierarchical nature of the model, models were compared using deviation = −2 (log likelihood ratio), and the best-fit model was determined by taking the model with the lowest deviance. The variance inflation factor (VIF) was used to detect multicollinearity, and the mean value of the VIF of the final model was 2.1.

#### Parameter estimation method

**The fixed effects (a measure of association)** were used to estimate the association between the likelihood of the prevalence of children living with non-biologic parents and explanatory variables at both individual and community levels. The association between dependent and independent variables was assessed, and its strength has been presented using an adjusted odds ratio (AOR) and 95% confidence intervals with a *p* < 0.05. Hence the log of probability of children living with non-biologic parents was modeled using a two-level multilevel by using the Stata syntax xtmelogit (10).


$$\begin{array}{l} logit(\pi ij)=log[\pi ij/(1-\pi ij)]=\beta 0+\beta 1xij+\beta 2xij\ldots .\\ \;+u0j+\;e0ij, \end{array}$$

Where πij: the probability of the ith child living with a non-biologic partner (1 – πij), the probability of young children not living with non-biologic parents, β0: intercept, βn: regression coefficient indicating that a unit increase in x can cause a unit increase in the probability of children living with non-biologic parents, Xij: independent variables u0j: community-level error (the effect of community on the lives of children with non-biologic parents); e0ij: individual-level errors. The clustered data nature and the within and between community variation were taken into account, assuming each community has a different intercept (βn) and fixed coefficient (β0) (11).

#### Random effects (a measure of variation)

Variation of the outcome variable or random effects was assessed using the proportional change in variance (PCV), intra-class correlation coefficient (ICC), and median odds ratio (MOR) (12, 13).

The ICC shows the variation of children living with non-biologic parents due to community characteristics, which was calculated as: **ICC** = **Vc**/(**Vc**+**3.29**), where **Vc** is the variance of the cluster (14). The higher the ICC, the more relevant the community characteristics are for understanding individual variation in children living with non-biologic parents.

MOR is the median value of the odds ratio between the areas with the highest number of children living with non-biologic parents and the area with the lowest number of children living with non-biologic parents when randomly picking out two children from two clusters, which was calculated as: $MOR=e^{0.95\sqrt{Vc}}$, where **Vc** is the variance of the cluster. In this study, MOR shows the extent to which the individual probability of children living with non-biologic parents is determined by the residential area (15).

Furthermore, the PCV illustrates how different factors account for variations in the prevalence of children living with non-biologic parents and is computed as: **PCV** = **Vnull** − **Vc/(Vnull)**, where Vc is the cluster-level variance and Vnull is the variance of the null model (16).

### Ethical approval and consent

Permission to access the data set was obtained from the MEASURE DHS International Program after the purpose of the analysis was communicated and approved by the DHS program. The original EDHS data were collected in accordance with international and national ethical guidelines. Ethical clearance for original DHS was provided by the Ethiopian Public Health Institute Review Board, the National Research Ethics Committee at the Ministry of Science and Technology, the Institutional Review Board of ICF International, and the United States Center for Disease Control. The data was only used for the purpose of this study and not shared with third parties. At the time of data collection for original (44), mothers and caregivers provided written informed consent, and data were collected anonymously. The data was fully available on the full DHS website (http://www.measuredhs.com/).

## Result

### Prevalence of children not living with biological parent in Ethiopia

**Figure 1:**
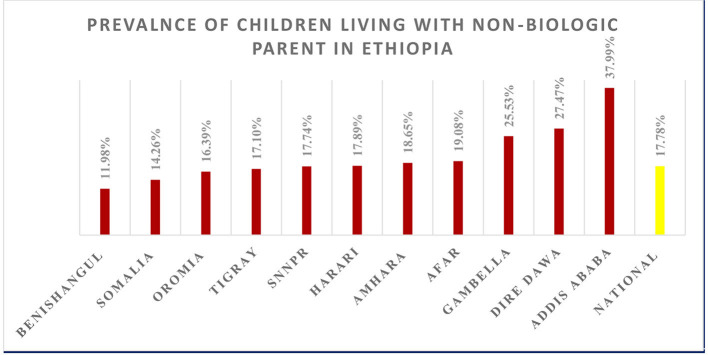
Regional prevalence of children living with non-biologic parent in Ethiopia.

The prevalence of children under 18 years old (12) not living with a partner in Ethiopia was 17.58 (95% CI, 17.22–17.95), with the lowest prevalence observed in Benishangul Gumuz (11.98%), while the highest prevalence was notable in Addis Ababa (37.99%) (Figure 1).

### Socio-demographic, individual, and community characteristics of children not living with their biologic parents in Ethiopia

A total weighted sample of 41,884 children under the age of 18 was included in the study. The majority of (88.65%) under-five children in the household were less than three in number. 17.5% of children reside in a mother-headed household, and 63.7% of children reside in a father-headed household. More than three-fourths (79.94%) of children reside in rural regions. More than half (50.41%) of children live in low-socio-economic households. More than half (50.77%) of children were from the community and had no formal education (Table 1).

**Table 1:** Socio-demographic, individual and community characteristics of children not living with their biologic parents in Ethiopia.

**Variable**	**Response**	**Frequency (*n*)**	**Percent (%)**
Child sex	Male	5,512	50.96
	Female	5,305	49.04
Orphan	Yes	3,087	8.16
	No	37,743	91.84
No of under five children	≤ 3	35,147	88.65
	< 4	4,500	11.35
Parent education	No education	20,128	50.77
	Educated	19,519	49.23
Hx of Death in the family	Yes	193	0.49
	No	7,252	99.51
No of household	< 5 ≥ 6	15,043 24,604	37.94 62.06
Family structure	Formal	23,493	59.26
	Non-formal	16,154	40.74
Household head sex	Male	29,557	74.55
	Female	10,090	25.45
Birth order	First	11,877	19.55
	Second and above	17,720	80.45
Wealth index	Poor	19,988	50.41
	Middle	5,580	14.07
	Rich	14,079	35.51
**Community level factors**			
Place of residence	Urban	7,953	20.06
	Rural	31,694	79.94
Community illiteracy level	High	21,443	54.08
	Low	18,204	45.92
Community poverty level	High	20,040	50.55
	Low	19,607	49.45
Region	Tigray	3,915	9.87
	Afar	3,206	8.09
	Amhara	3,963	10
	Oromia	5,719	14.42
	Somalia	5,175	13.05
	Benshangul	3,186	8.04
	SNNP	5,195	13.1
	Gambela	3,003	7.57
	Harar	2,181	5.5
	A.A	1,837	4.63
	Dire Dawa	2,267	5.72

A.A., Addis Ababa; Hx, History.

### Random effect analysis and model fit statistics

We conducted a community-level variation assessment using both ICC and MOR. As the null model ICC illustrates 12.7% variation of children living with non-biologic parents was attributable to differences between clusters. The median odds ratio in the null model also revealed that children living with their non-biologic partner were 1.93 variables between high and low clusters. In addition, PCV in the final model showed 41.66% of the variation in children residing with non-biologic parents was attributed to individual and community-level factors. For model comparison, deviance was utilized. The best-fit model, the final model (III), was chosen based on its lowest deviance (Table 2).

**Table 2:** Random effect and model fit statistics of children resides in non-biologic parent.

**Parameter**	**Null model**	**Model I**	**Model II**	**Model III**
Variance	0.48	0.84	0.22	0.68
ICC	12.73	20.34	6.27	17.13
MOR	1.93	2.39	1.56	2.19
PCV	**Reference**	75.0 %	54.16%	41.66 %
**Model comparison**				
LLR	−18,443.66	−1,453.06	−18,524.6	−1,434.35
Deviance	36,887.32	2,906.12	37,049.2	2,868.7

ICC, Intra Cluster Correlation Coefficient; MOR, Median Odds Ratio; PCV, Proportional Change in Variance; LLR, Log-likely-hood Ratio.

### Factors associated with children living with non-biologic parent in Ethiopia

In the final multi-variable analysis, factors including parental death, household lack of formal family structure, household head sex, birth order, residence of the child, and children from high community poverty levels were significantly associated with children living with non-biological parents.

Being born to a deceased parent increases the odds of children living with a non-biologic parent by four times (AOR = 4.17; 95% CI, 2.40–7.25) as compared to their counterparts. First-born children had an increased likelihood of living with non-biologic parents by eight times (AOR = 8.22; 95% CI = 39.17) as compared to second and above-order born children. Similarly, being from a household lacking formal family structure (no husband or wife) in the household increases the odds of children living with a non-biologic parent by 18 times (AOR = 8.6; 95% CI, 12.59–27.5) as compared to children from formal family structure. Being a female-headed household increases the likelihood of children living with a non-biologic parent by three times (AOR = 3.43; 95% CI, 2.65–4.43) as compared to a male-headed household. From community-level factors, being in rural residence increases the likelihood of children not living with biologic parents by 94% (AOR = 1.94; 95% CI: 1.23–3.06) as compared to children who reside in urban areas. Similarly, children from high community poverty levels were 25% (AOR = 1.25, 95% CI, 1.01–1.75) more likely to live with non-biologic parents as compared to children in low community poverty levels (Table 3).

**Table 3:** Factors associated with children live in non-biologic parent.

**Individual level factors**	**Response**	**Model II AOR (95 % Cl)**	**Model III AOR (95 % Cl)**	**Model IV AOR (95 % Cl)**
Child sex	Male	4.24 (2.40–7.49)		0.94 (0.76–1.15)
	Female	1		1
Parent died	Yes	0.78 (0.37–1.63)		4.17 (2.40–7.25)^*^
	No	1		1
No of under five children	< 3	1		1
	>4	1.14 (0.85–1.52)		1.30 (0.97–1.73)
Parent education level	No education	4.95 (0.65–37.63)		6.22 (4.8–8.33)
	Educated	1		1
Hx of Death in the family	Yes	0.78 (0.37–1.63)		0.7 (0.36–1.48)
	No	1		1
No of household	< 5	0.07 (0.06–0.10)		0.08 (0.06–1.1)
	≥6	1		1
Family structure	Formal	1		1
	Non-formal	20.6(13.93–30.5)		8.60 (6.2–12.3)^*^
Household head sex	Male	1		1
	Female	3.04 (2.35–3.92)		3.43 (2.65–4.43)^*^
Birth order	First	8.2 (6.1–13.9)		8.22 (6.31–9.17)^*^
	Second and above	1		1
Wealth index	Poor	1.36 (1.04–1.77)		1.00 (0.69–1.44)
	Middle	2.03 (1.43–2.88)		
	Rich	1		1.45 (0.99–2.14) 1
**Community level factors**				
Place of residence	Urban		1	1
	Rural		1.86 (1.61–2.14)	1.94 (1.23–3.06)^*^
Community illiteracy	High		1.28 (1.14–1.43)	1.29 (0.94–1.76)
	Low		1	1
Community poverty	High		1.13 (0.99–1.28)	1.25 (1.01–1.75)^*^
	Low		1	1
Region	Tigray		1	1
	Afar		1.42 (1.14–1.77)	1.06 (0.60–1.88)
	Amhara		1.08 (0.88–1.32)	0.97 (0.54–1.74)
	Oromia		1.06 (0.87–1.29)	1.16 (0.68–1.97)
	Somalia		0.93 (0.75–1.15)	0.49 (0.27–0.1.2)
	Benshangul		0.71 (0.56–0.89)	0.48 (0.24–1.14)
	SNNP		1.12 (0.92–1.37)	1.14 (0.65–1.99)
	Gambela		1.35 (1.09–1.67)	1.26 (0.71–2.22)
	Harar		0.92 (0.73–1.170)	1.25 (0.63–2.46)
	A.A		1.68 (1.33–2.13)	1.07 (0.56–2.04)
	Dire Dawa		1.51 (1.20–1.90)	1.38 (0.74–2.60)

A.A., Addis Ababa; AOR, Adjusted odds ratio.

^*^statistically significant (p < 0.05); CI, Confidence interval.

## Discussion

Family-unbound children's living arrangement is a common practice in low-income countries. In Ethiopia, children living with a non-biologic partner are increasing secondary to poverty, economic challenges, extended family norms, religious influence, ongoing civil war, drought, internal displacement, access to health care/education, and urban migration secondary to economic constraints.^7^ Accordingly, this study found that the magnitude of children under 18 years old (12) living with a non-biologic parent in Ethiopia was 17.58% (95% CI, 17.22–17.95). This finding is higher than the previous DHS data survey (45) (see text footnote 7). The possible explanation could be Ethiopian population growth continuously every year. In addition, Ethiopia's drought and political unrest have been becoming worse each year. Furthermore, large-scale disruption, such as internal displacement and the impact of ongoing violence, increases the incidence of non-biologic living arrangements in Ethiopia. As a result, the government should prioritize the issue in order to minimize the load through collaboration with domestic and foreign partners.

This study finding is higher than the study on Uganda (15.7%), India, Nepal (5–8%),^8^ and Rwanda (15%).^9^ The possible explanation could be Ethiopia had a higher fertility rate, drought, famine, migration, internal displacement, and changing social structure responsible for family unbound children (17, 18).^10^ In addition, Ethiopia had a strong cultural family tradition of informal caring, and the country's history of civil war and ethnic conflict had contributed considerably to family breakdown. Furthermore, unlike Rwanda and India, which have larger government or NGO-led child welfare programs, Ethiopia is mainly reliant on traditional caring systems that are less structured and more difficult to supervise.

However, this study's findings are lower than those of Malawi (18%), Lesotho (20%), Zimbabwe (19%), and Burundi (15%) (see text footnote 8).^11^ The possible explanation could be that these countries had a high fertility rate, a high poverty rate, a high HIV/AIDS infection rate, and food insecurity that led children to orphanhood and live separately from their parents. AIDS is the single largest factor behind the high orphan rate in Malawi (19–21). In addition, these countries have a strong tradition of fostering and extended family care, which intended to support vulnerable children often results in children living with non-biologic partners, but Ethiopia has a tradition of extended family care; however, its cultural practices emphasize keeping children within the direct family or very close kin. This, combined with Ethiopia's relatively lower orphan rate, results in fewer children being placed with non-biologic parents compared to Malawi and Burundi (22).

A female-headed household increases the likelihood of children living with non-biologic parents as compared to a male-headed household. This is supported by the study done in Nigeria (23). This is due to the fact that females in many societies are seen as primary caregivers, making them more likely to take an orphaned child, especially when family ties exist; widowed or single women might step into caregiving roles for relatives' children. Widowed women in developing countries often inherit caregiving duties for children from deceased relatives or friends. Female-headed households often are sometimes formed by economic hardship or social displacement like conflict, migration, drought, and illness like HIV/AIDS (24–27). All the above-mentioned realities predispose female-headed households to harbor more orphan and non-biologic children. Therefore, female-headed households shall be supported by relevant agencies since they have financial and social challenges. Tailored support for these households, including financial aid, education, and community support, can help alleviate the burden they carry while ensuring a better outcome for orphaned children. In addition, gender norms in many nations consider women's as a primary care giver, often leading female headed household to assume responsibility for orphan and non-biologic children. These gender norms reflect societal expectation of women as nurturers but also impose significant emotional and economic burden. As a result, policy makers and other entities shall support these households and amend traditional gender roles by promoting shared care giving responsibility and addressing structural inequality.

According to this study, birth order is significantly associated with children not living with their biological partner. Thus, children in first order were subsequently increasing the odds of not living with their parents compared to those in second and above. The possible explanation could be that early paternal death contributes to this difference. If parents die at an early age, their children are forced to be orphans or live with other non-biologic parents. In addition, separation, migration, being in prison, or other factors become more pronounced as relations go among couples (28, 29).

As compared to children living in urban areas, children who reside in rural areas had increased odds of living with non-biologic parents. Nonetheless, the majority of orphan-to-non-orphan differences tend to be larger in metropolitan regions, and orphan prevalence rates are disproportionately higher there; our study revealed contradictory evidence. A plausible explanation is that work represents a significant life transition and an integral part of the expectations for children in rural Ethiopia. Youngsters in these regions often engage in work for other households, both to provide financial support to their struggling families and to foster their own personal growth (21, 30, 31). In addition, greater poverty in rural areas, limited educational opportunity, cultural tradition, and agricultural labor in rural areas forced children to live with non-biologic parents on the rural side of Ethiopia.

Children whose parents died were more likely to live with non-biologic parents as compared to children whose parents were alive. This is supported by other studies (32–34). The possible explanations include the direct and immediate impact of losing a parent, a decline in financial assistance, and a shift in social and emotional support. Children left without a caregiver, without emotional or financial support from their parents, are forced to join another family member. Moreover, following the death of a parent, some children live with their remaining parent, while others live with someone else, such as a stepmother, stepfather, grandmother, aunt, uncle, sibling, foster parent, or adoptive parent (35). In addition, parent death reduces household income, shift household labor demand, and parent love (36, 37).

Children from a high community poverty level were more likely to live with non-biologic parents as compared to children from a low community poverty level. This is supported by the study done in different parts of the world (34, 38). This could be due to non-parental care often co- occurs with poverty (5, 39). Furthermore, children who experience poverty may be forced to leave their family and seek refuge, food, or education with another family. Additionally, poverty is a swamp that complicates multiple aspects of caregiving and other factors of parentlessness worsened by poverty (39).

Households lacking formal family structure increase the odds of children living with non-biologic parents. This is supported by the study done in Zambia, and south Africa (40, 41). The possible explanation could be that family stability is thought to be crucial for the wellbeing of children and adolescents because it promotes healthy attachment (42). However, more parentless youngsters will be drawn to the home if there is a disruption in the family (either the husband or the wife missed). A complex family structure in which children co-reside with stepparents and stepsiblings increases the probability of non-parent children included in the family; probably, children of the whole family will aggregate from the antecedent stepfather's home. Most of the time, orphans live with their relatives or other family members; this arrangement is typically informal, but for youngsters without parents, it is a suitable type of care. Orphans who live with family members or extended family, however, are more likely to experience prejudice and discrimination from other members of the home. According to a number of studies, these prejudices and discriminations raise the possibility of being left out, abused, or exploited (43).

Since it is based on data from a nationwide survey, the study has the potential to help programmers and policymakers develop effective interventions at the national level. But the DHS is primarily dependent on respondents' self-reports, so there is a chance of recall bias in this study that leads to underrepresentation. In addition, there might be non-representative for marginalized or hard-to-reach population groups. Furthermore, due to the DHS nature of the data, important variables related to living arrangements like social determinants, health system, urban-rural dichotomy, and aggregated data challenges lead to overestimation or underestimation of children living with non-biologic parents. Therefore, future research addressing these limitations shall be conducted, particularly primary research on the living arrangements of children in Ethiopia, considering urban-rural differences from the woreda to the national level.

## Conclusion

A significant proportion of children live with a non-biologic parent in Ethiopia. The study discovered that both individual and community-level factors were associated with children not living with non-biologic parents based on existing data. In our study, being an orphan, birth order, lack of a normal family structure, a female-headed household, rural residence, and a low socio-economic community were factors associated with children not living with their biological parents. Therefore, the government and other stakeholders shall give special attention to orphan children, female headed household, rural children. In addition the government shall bring and long term economy improvement in the household at lower and at national level at largest.

## Recommendations

Policymakers should implement orphan care programs, raise awareness on family structure, community-based poverty alleviation through economic development initiatives, and empowering women, and promote multi-sectorial collaboration with stakeholders. The federal ministry of health should implement a holistic health program focusing on parental death and poverty, support female-headed households through skill development, and implement a comprehensive sexual and reproductive health education program to decrease maternal mortality. The government should empower female headed through social support program, economic and educational support, promoting initiatives to formalize informal family structure such as legal recognition of guardian ship for household lacking formal family structure, develop community based child welfare system for rural children, and increase household income and the government should plan and work toward reducing poverty in the long ran.

In addition, the government shall work in collaboration with other stakeholders to empower women through education and economic support, legalize informal family structure to formal family structure, help disadvantageous rural children through the community child welfare system, and to bring economic development in the long term in the country.

Future researchers shall do a qualitative study of why children are forced to live with non-biologic parents apart from parental death.

## Data Availability

The datasets used and/or analyzed during the current study are available from the corresponding author on reasonable request.

## Footnotes

^1^ https://www.unicef.org/

^2^ https://data.unicef.org/

^3^ https://www.unicef.org/ethiopia/understanding-situation-children

^4^ https://latitude.to/map/et/ethiopia

^5^ https://dhsprogram.com/data/Guide-to-DHS-Statistics/index.cfm

^6^ https://dhsprogram.com/data/Guide-to-DHS-Statistics/Children%E2%80%99s_Living_Arrangements_and_Orphanhood.htm

^7^ https://bettercarenetwork.org/sites/default/files/Ethiopia%20DHS%202011-%20%20Children%E2%80%99s%20Care%20and%20Living%20Arrangements.pdf

^8^ https://www.unicef.org/media/90761/file/India-2019-COAR.pdf

^9^ https://www.unaids.org/en/resources/documents/2020/global-aids-report

^10^ https://www.worlddata.info/country-comparison.php?country1=ETH&country2=UGA

^11^ https://dhsprogram.com/pubs/pdf/FR319/FR319.pdf
